# Toll-Like Receptor- and Protein Kinase R-Induced Type I Interferon Sustains Infection of *Leishmania donovani* in Macrophages

**DOI:** 10.3389/fimmu.2022.801182

**Published:** 2022-01-28

**Authors:** Bruna T. Dias, Amy Goundry, Aislan C. Vivarini, Tatiana F. R. Costa, Jeremy C. Mottram, Ulisses G. Lopes, Ana Paula C. A. Lima

**Affiliations:** ^1^ Instituto de Biofisica Carlos Chagas Filho, Universidade Federal do Rio de Janeiro, Rio de Janeiro, Brazil; ^2^ York Biomedical Research Institute, Department of Biology, University of York, York, United Kingdom

**Keywords:** *Leishmania*, interferon, PKR, TLR, neutrophil elastase (NE), ecotin

## Abstract

*Leishmania donovani* is a protozoan parasite that causes visceral leishmaniasis, provoking liver and spleen tissue destruction that is lethal unless treated. The parasite replicates in macrophages and modulates host microbicidal responses. We have previously reported that neutrophil elastase (NE) is required to sustain *L. donovani* intracellular growth in macrophages through the induction of interferon beta (IFN-β). Here, we show that the gene expression of IFN-β by infected macrophages was reduced by half when TLR4 was blocked by pre-treatment with neutralizing antibodies or in macrophages from *tlr2* ^-/-^ mice, while the levels in macrophages from *myd*88^-/-^ mice were comparable to those from wild-type C57BL/6 mice. The neutralization of TLR4 in *tlr2* ^-/-^ macrophages completely abolished induction of IFN-β gene expression upon parasite infection, indicating an additive role for both TLRs. Induction of type I interferon (IFN-I), OASL2, SOD1, and IL10 gene expression by *L. donovani* was completely abolished in macrophages from NE knock-out mice (*ela2* ^-/-^) or from protein kinase R (PKR) knock-out mice (*pkr* ^-/-^), and in C57BL/6 macrophages infected with transgenic *L. donovani* expressing the inhibitor of serine peptidase 2 (ISP2). Parasite intracellular growth was impaired in *pkr* ^-/-^ macrophages but was fully restored by the addition of exogenous IFN-β, and parasite burdens were reduced in the spleen of *pkr* ^-/-^ mice at 7 days, as compared to the 129Sv/Ev background mice. Furthermore, parasites were unable to grow in macrophages lacking TLR3, which correlated with lack of IFN-I gene expression. Thus, *L. donovani* engages innate responses in infected macrophages *via* TLR2, TLR4, and TLR3, *via* downstream PKR, to induce the expression of pro-survival genes in the host cell, and guarantee parasite intracellular development.

## Highlights

*Leishmania donovani* are pathogenic protozoa that cause deadly disease. The parasites are transmitted by the bite of infected phlebotomine insects and disseminate to the liver, spleen, and the bone marrow, where they cause inflammation and tissue destruction. The parasite multiplies in cells of the host immune system, the macrophages, and modulate host defenses to propagate infection. It is important to understand how the parasites can modulate host defense to improve disease treatment. Here, we describe how *L. donovani* takes advantage of host anti-microbial receptors and innate responses in order to sustain infection inside macrophages.

## Introduction

Visceral leishmaniasis (VL) is a deadly disease caused by the parasitic protozoa *Leishmania donovani* or *Leishmania infantum*. The parasites are transmitted to mammals through the bite of infected phlebotomines (sand fly) and disseminate from the wound site to the liver and spleen, provoking anemia, hepato- and splenomegaly, tissue destruction, and, in some cases, hemorrhaging (1, 2). Infective parasites are flagellated metacyclic promastigotes that can be taken up by phagocytes at the site of infection. In macrophages, they differentiate to amastigotes that multiply inside a parasitophorous vacuole generated from the phagosome through the fusion/recruitment of elements from different endosomal compartments (3). In experimental VL, parasites replicate in splenic macrophages of the red pulp and of the marginal zone or in liver macrophages (Kupffer cells) and have evolved to downmodulate macrophage responses (4, 5). A variety of molecular pathways related to inhibition of innate responses by the parasite have been identified, as well as the hijacking of immune responses to favor parasite survival and/or replication [reviewed in (6). While Toll-like receptor 4 (TLR4) has been identified as important for the induction of interferon gamma (IFN-γ), tumor necrosis factor (TNF), and inducible nitric oxide synthase (iNOS) in the liver of *L. donovani*-infected mice, controlling parasite burden, TLR2 was associated with the promotion of infection (51). At the single-cell level, parasite surface lipophosphoglycans (LPG) stimulate TLR2 in macrophages, which was reported to be involved in the modulation of the kinetics of phagosome maturation, allowing parasite differentiation and survival (7). LPG is also required to protect the parasites from the microbicidal activity from neutrophil extracellular traps (8). The adaptation to cope with host cell microbicidal responses varies among different *Leishmania* species. As an example, *L. amazonensis*, a species associated with cutaneous disease, employs the LPG-TLR2 pathway to stimulate downstream routes typically associated to anti-viral responses, i.e., the activation of the double-stranded RNA-dependent protein kinase R (PKR), and downstream induction of type I interferon (IFN-I) expression (9, 10) PKR activation and the upregulation of its expression, leading to IFN-β production, is required for the concomitant production of IL10, and sustained parasite multiplication in infected cells (10). In contrast, IFN-β has leishmanicidal activity against *L. major* (11). We have previously shown that *L. major* prevents the induction of such innate responses in macrophages by inactivating cell surface neutrophil elastase (NE) *via* the endogenous parasite serine peptidase inhibitor ISP2, which, otherwise, controls parasite development (12, 13). We found that in the absence of ISP2, during parasite phagocytosis, NE triggers a TLR4-dependent pathway that leads to PKR-mediated induction of IFN-I and control of parasite replication (13). Lack of ISP2 also impacts long-term parasite survival in infected mice, mainly due to sustained recruitment of iNOS^+^ monocytes to the lesion site and higher local IFN-γ levels (14).

More recently, we found that *L. donovani*, similarly to *L. amazonensis*, takes advantage of innate responses, requiring IFN-β to develop in murine macrophages (15). The induction of IFN-β gene expression was partially dependent on the NE-TLR4 pathway that ensured a threshold of IFN-β levels to sustain parasite survival/replication (15). Those observations led to the hypothesis that during *L. donovani* infection of macrophages, additional components are required for the induction of IFN-β and permissive parasite growth. Here, we show that both TLR4 and TLR2 act in conjunction in macrophages to promote IFN-β gene expression upon infection, and that this is entirely dependent on PKR. Furthermore, we found that TLR3 is required for the downmodulation of inflammatory responses in *L. donovani*-infected macrophages and parasite intracellular growth, connecting typical anti-viral innate responses to the promotion of parasite infection.

## Methods

### Parasites

Wild-type promastigotes of the *L. donovani* MW897 Sudanese strain were grown in hemoflagellate modified Eagle’s medium (designated HOMEM medium) supplemented with 10% heat-inactivated fetal calf serum (FCS) (Gibco) at 27°C. The transgenic line of *L. donovani* MW897 expressing *L. major* ISP2 (*L. donovani:*ISP2) was generated previously (15).

### Mice

The mice colonies were kept at the Laboratório de Animais Transgênicos (LAT) at the Universidade Federal do Rio de Janeiro (UFRJ; Rio de Janeiro, Brazil). PKR-knockout mice (*pkr* ^-/-^) in the 129Sv/Ev background were donated by Dr. Aristobolo Silva of the Universidade Federal de Minas Gerais (UFMG; Minas Gerais, Brazil). C57BL/6, *ela2* ^-/-^, 129Sv/Ev, and *pkr* ^-/-^ were bred in-house at LAT, and *myd88* ^-/-^, *tlr2* ^-/-^, and *tlr3* ^-/-^ mice in the C57BL/6 background were kindly donated by Dr. M Bellio (UFRJ), Dr. Marcelo Bozza (UFRJ), and Dr. Renata Meirelles Pereira (UFRJ), respectively. Female mice between 6 and 10 weeks were used in the experiments. All mice were handled according to protocols approved by the ethics committee (Comissão de Ética no Uso de Animais (CEUA) 034/15–UFRJ).

### Macrophage Infections

Peritoneal macrophages from C57BL/6, *ela2* ^-/-^, *myd88* ^-/-^, *tlr2* ^-/-^, *tlr3* ^-/-^, 129Sv/Ev, and *pkr* ^-/-^ mice were elicited by injection of 1% thioglycolate solution into the peritoneal cavity. An average of 2–3 mice for each genetic background were injected; after 3 days, the mice were euthanized and the peritoneal cells were collected through washing of the peritoneal cavity with 5 ml of ice-cold Roswell Park Memorial Institute (RPMI) 1640 medium (Sigma) and pooled. Cells were centrifuged at 300 × *g* for 10 min, washed with Hank’s buffered saline solution (HBSS), and then counted. Cells were plated in RPMI-10% FCS at different concentrations depending on the experiment and incubated overnight at 37°C and 5% CO_2_. After 24 h, the monolayers were washed with HBSS, and the adherent cells were used as follows.

For infection and survival assays, macrophages were plated at 4 × 10^5^ cells per coverslip in the wells of a 24-well tissue culture plate and infected with late-stage promastigotes (culture density of 3 × 10^7^/ml) at a 5:1 parasite:cell ratio in RPMI supplemented with 0.1% bovine serum albumin (BSA; Sigma) at 37°C and 5% CO_2_. After 3 h, cells were washed with HBSS to remove extracellular parasites. Macrophages were then either fixed with 70% methanol and Giemsa-stained at 3 h, or cultured in RPMI-10% FCS, until 24 h, 48 h, or 72 h after washing and then fixed and stained. The number of intracellular parasites was determined by counting at least 100 cells per replicate under a light microscope at 100× oil immersion objective. Where indicated in the figure legends, 200 ng/ml IFN-β (BioLegend) was added after the removal of extracellular parasites at 3 h and cultivated for 72 h in RPMI-10% FCS.

The 14M1.4 mouse stromal macrophage cell line [a gift from Paul Kaye, University of York, UK (16)] was cultivated in Dulbecco’s Modified Eagle’s medium (DMEM) (Thermo Fisher Scientific–Invitrogen) supplemented with 10% FCS at 37°C and 5% CO_2_. For infection experiments, cells were washed twice with phosphate-buffered saline (PBS), treated with 7 μg/ml mitomycin C (Sigma) for 3 h at 37°C, washed, and dissociated by scraping with Rubber policeman. Cells were collected by centrifugation, plated on glass coverslips as described above, and cultivated overnight before infection.

For protein extraction, 2 × 10^6^ cells per well were plated in 6-well plates and infected with late-stage promastigotes at a 10:1 ratio in RPMI-0.1% BSA at 37°C and 5% CO_2_ for 2 h. After this time, cells were washed with HBSS to remove extracellular parasites and incubated for 6 h or 24 h. To obtain the nuclear fraction, at the indicated times, cells were washed three times with ice-cold PBS and then lysed in a lysis buffer (20 mM HEPES, 10 mM NaCl, 1 mM EDTA pH 7.5, and 5 mM DTT), supplemented with Protease Inhibitor Cocktail (Sigma). The suspension was extracted on ice for 10 min and centrifuged at 15,682 × *g* for 5 min at 4°C. The pellet was resuspended in a buffer (20 mM HEPES, 400 mM NaCl, 1 mM EDTA, pH 7.5, 5 mM DTT, and 6.25% glycerol), supplemented with Protease Inhibitor Cocktail, and extracted on ice for 10 min by vortexing every 2 min. The suspension was centrifuged at 15,682 × *g* for 8 min at 4°C and the supernatant was collected as the nuclear fraction. The total protein was quantified using the Pierce BCA Protein Assay (Thermo Fisher Scientific) and 50 μg of protein was diluted in sample buffer (10% glycerol, 100 mM β-mercaptoethanol, 2% SDS, 50 mM Tris-HCl, pH 6.7, and 0.1% bromophenol blue) and then boiled for 5 min before use in Western blotting. To obtain total cell lysate, cells were infected for 1 h in RPMI-0.1% BSA and then washed three times with ice-cold PBS. Where indicated, macrophages were incubated with 100 nM Toll-like receptor 4 (TLR4) inhibitor, TAK-242 (Resatorvid; MedChemExpress), or 10 µM of the irreversible NE inhibitor III (NEI; Calbiochem) for 15 min prior to addition of the parasites. Cells were lysed in 100 µl of CellLytic M (Sigma) supplemented with Protease Inhibitor Cocktail and PhosSTOP Phosphatase Inhibitor Cocktail (Merck) on ice and centrifuged at 10,000 × *g* for 10 min, and the supernatant was collected.

For RNA extraction, 3 × 10^6^ cells per well were plated in a 6-well plate and infected with late-stage promastigotes at a 10:1 ratio in RPMI-0.1% BSA at 37°C and 5% CO_2_. After 2 h or 3 h, as indicated in the figure legends, extracellular parasites were removed by washing. In the experiments of receptor neutralization, 10 μg/ml anti-mouse TLR4 neutralizing antibodies (CD284/MD2 complex clone MTS510; Thermo Fisher Scientific) or 10 μg/ml IgG (IgG2aK control clone eBR2a; Thermo Fisher Scientific) were incubated for 30 min with the macrophages in RPMI-10% FCS and then washed twice before infection with the parasites in RPMI-0.1% BSA at 37°C and 5% CO_2_. Where indicated in the figures, poly I:C was used at 25 µg/ml. After removal of the parasites, cultures were incubated until 6 h in RPMI-0.1% BSA. At the indicated times, total RNA was extracted with the RNeasy Mini Kit (Qiagen) using a 1-ml syringe to initially lyse the cells.

### Immunofluorescence

Peritoneal macrophages from C57BL/6 or *ela2* ^-/-^ elicited by thioglycolate as described above were cultured overnight on glass coverslips in 24-well plates in RPMI-10%-FCS. Monolayers were washed with HBSS and incubated with late-stage promastigotes at a 20:1 parasite:macrophage ratio for 15 min in RPMI-0.1% BSA at 37°C and 5% CO_2_. Extracellular parasites were removed by repeated washing with HBSS and cultures were incubated in RPMI-10% FCS at 37°C and 5% CO_2_ up to the indicated times in the figure legends. At the time points, monolayers were washed and fixed in 4% methanol-free paraformaldehyde (Thermo Fisher Scientific) for 20 min at room temperature. Cells were then washed in PBS, permeabilized in PBS-0.1% saponin for 1 h, washed again, and incubated with 0.1 M glycine for 10 min. Cells were blocked in PBS-0.01% saponin containing 0.1% BSA for 1 h and then incubated with monoclonal anti-Rab7 (Abcam, ab137029) at a 1:500 dilution in PBS-0.1% BSA for 2 h at room temperature, followed by three washes with PBS and incubation with Cy3-goat anti-rabbit IgG (Invitrogen, A10520) at a 1:1,000 dilution for 1 h. After washes, coverslips were mounted using Fluoroshield with DAPI (Immunobioscience by Sigma) on slides and sealed with nail polish. For TLR3 and IRF7 labeling, macrophages were infected at a 10:1 parasite:macrophage ratio for 3 h in RPMI-0.1% BSA, extracellular parasites were removed by repeated washing with HBSS, and cultures were fixed or incubated in RPMI-10% FCS at 37°C and 5% CO_2_ until 24 h, 48 h, or 72 h before fixation and preparation for IFA as above. Antibody dilutions were as follows: rabbit monoclonal anti-TLR3 (Abcam, ab62566) at 1:200, rabbit monoclonal anti-IRF7 (Abcam, ab62505) at 1:100, and the Cy3-goat anti-rabbit IgG secondary antibody (Invitrogen, A10520) at 1:2,000 dilution. Images were acquired on a Cell Observer spinning disk confocal microscope from Zeiss. Each sample was sectioned from the bottom to top at 0.48 μm for each slice. The slices were compressed to the maximum intensity projection (MIP) in a single image using the Zen Blue software.

### Western Blotting

Protein samples of the nuclear fraction or total cell lysate were separated by 11% SDS-PAGE, and the proteins were transferred onto a PVDF membrane. The membrane was blocked in Tris-buffered saline with 0.05% Tween 20 (TBS-T) together with 9% w/v nonfat dry milk for 1 h at room temperature. Membranes were washed 3 times for 5 min each with TBS-T. Antibodies to IRF3 (Abcam, ab238521) were added to the membranes and incubated for 2 h. After 3 washes, an anti-rabbit secondary antibody coupled to horseradish peroxidase (HRP) was used at a 1:4,000 dilution and incubated for 2 h. Antibodies to lamin A/C (Monoclonal clone 4C11; Sigma, SAB4200236) at 1:10,000 dilution were used for the loading control of the nuclear fraction. Anti-phospho-PKR (Thr451) Antibody (07–886 Merck Millipore) was used at 1:500 dilution in 5% w/v BSA, 1× TBS, 0.1% Tween 20 at 4°C with gentle shaking overnight, followed by goat anti-rabbit horseradish peroxidase-conjugated IgG (sc-2030, Santa Cruz Biotechnology).

### Quantitative PCR Assays

cDNA was synthesized using 1 μg of RNA and the Improm Kit (Promega). Real-time quantitative PCR (qPCR) assays of first-strand cDNA were performed with Step One (Thermo Fisher Scientific) and Sybr Green (Promega). The expression ratios were computed *via* the ΔΔCt method. The primers used are listed in **Supplementary Table 1**.

### *In Vivo* Infections

In total, 3 × 10^7^ stationary-phase promastigotes in 100 µl of PBS were injected in the retro-orbital cavity of 129Sv/Ev or *pkr* ^-/-^ mice. After 7 days, the mice were euthanized, and the spleens and livers were collected. The total body weights and organ weights were used to calculate the organ mass/g (**Supplemental Figure 1**) and were not significantly different between experimental groups. The spleens and livers were ruptured to homogeneity with the back of a syringe through a nylon membrane or cell strainer in HOMEM medium (Thermo Fisher Scientific) supplemented with 10% FCS. Cell homogenates were submitted to serial dilutions in 96-well plates and cultivated for 7 days at 27°C for parasite burden analysis. Spleens were ruptured in 2 ml of HOMEM-FCS and 500 μl of homogenates were taken to perform serial dilutions (undiluted, 1:10, 1:100, 1:1,000). Aliquots of 10 μl of the well with the highest dilution containing parasites were taken and the numbers of parasites were counted in the Neubauer chamber. Livers were ruptured in 3 ml of HOMEM-FCS and 500 μl of cell homogenates were taken to perform 3× serial dilutions (12 times sequentially). The parasite numbers were calculated by using the highest dilutions with parasites multiplied by the dilution factors.

### Statistical Analyses

Statistical analyses were performed using Prism 7.0 (GraphPad Software, La Jolla, CA, USA). The qPCR data were analyzed by one-way ANOVA using the Bonferroni posttest at a significance level of 5% and the macrophage infection data were analyzed by two-way ANOVA with Sidak’s multiple comparisons test. For the parasite burdens in the organs, data were analyzed by Student’s *t*-test.

## Results

**Figure 1:**
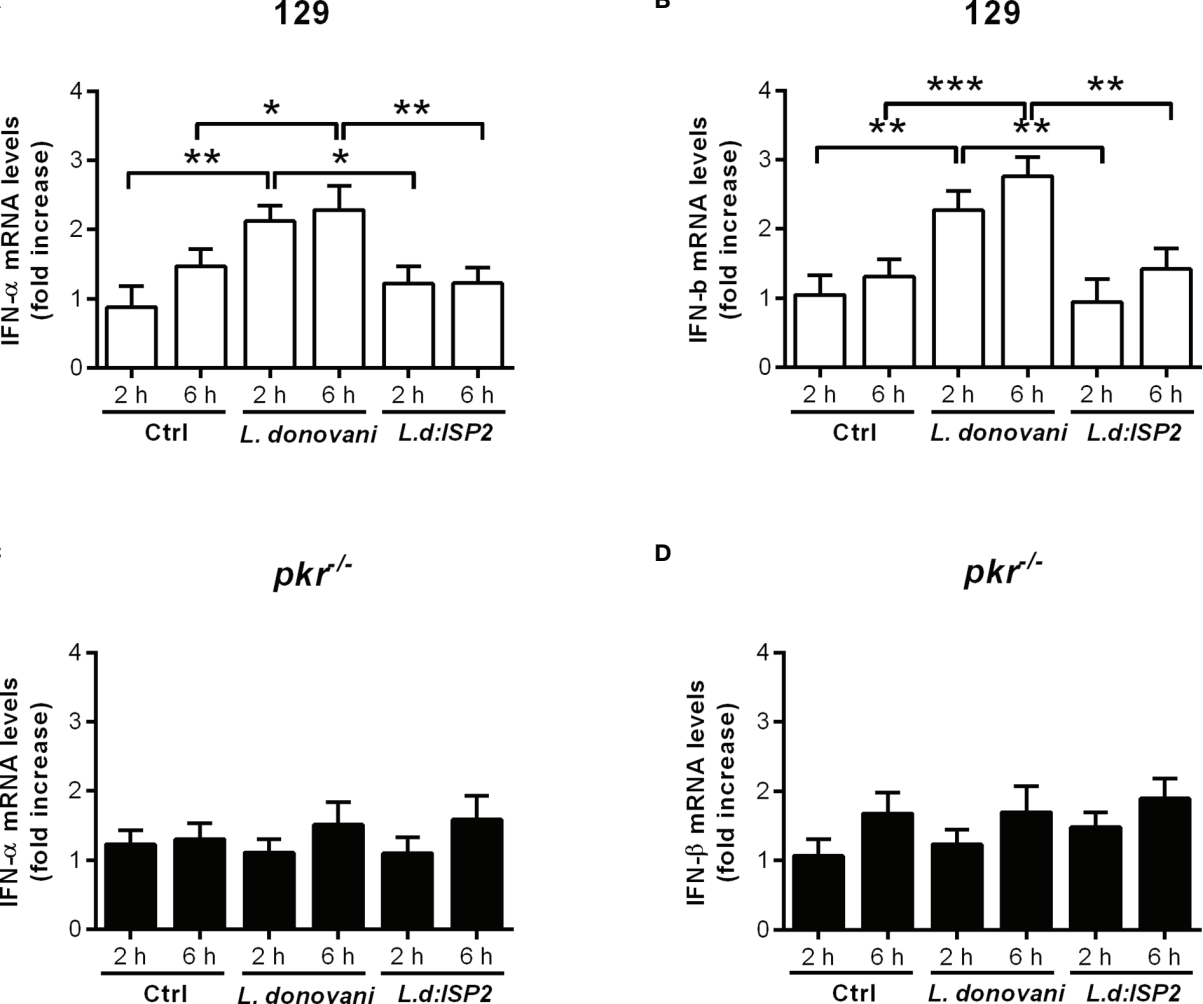
PKR is required for the induction of type I IFN in macrophages infected by *L. donovani*. Thioglycolate-recruited macrophages from 129Sv/Ev and *pkr* ^-/-^ mice were infected with stationary-phase promastigotes of *L. donovani* wild-type or *L. donovani* expressing ISP2 (*L.d:ISP*2) at a 10:1 parasite:macrophage ratio for 2 or 6 h. Total RNA was extracted, and cDNA was made and used as a template in qPCR for the determination of the relative mRNA levels for IFN-α **(A, C)** or IFN-β **(B, D)**. Uninfected cells were used as a control (Ctrl). The experiment was repeated 2 independent times. The graphs show representative experiments, with the means ± SD of the triplicate technical replicates. Statistical significance was assessed using one-way ANOVA with Bonferroni posttest. *p < 0.05, **p < 0.01, ***p < 0.001. Differences between the same time point or the same group are shown.

**Figure 2:**
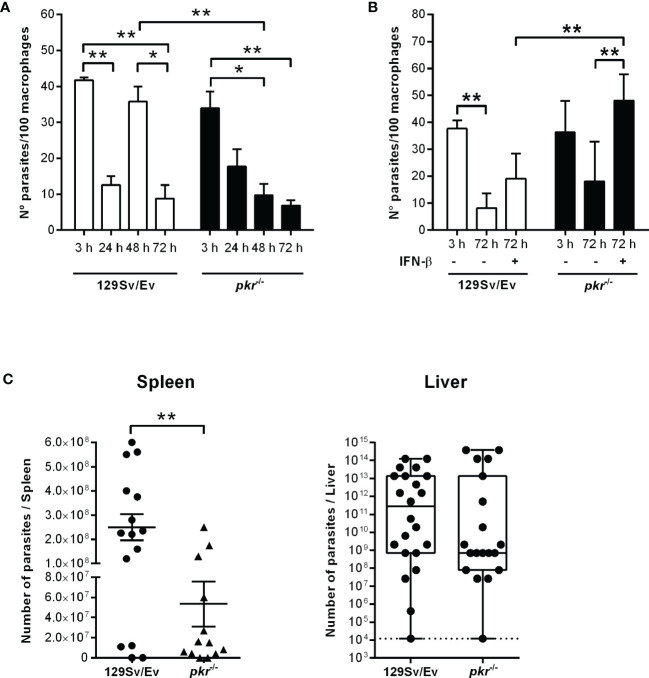
PKR is required for sustained parasite burdens in macrophages and in infected mice. **(A, B)** Peritoneal thioglycolate-recruited macrophages from 129Sv/Ev and *pkr^-/-^* mice were cultivated on glass coverslips overnight in RPMI-FCS and then washed and infected with late-stage promastigotes of *L. donovani* at a 5:1 parasite:macrophage ratio for 3 h at 37°C in RPMI-BSA. After 3 h, the monolayers were washed with HBSS to remove extracellular parasites, fixed with methanol and Giemsa-stained, or further incubated for 24 h, 48 h, or 72 h in RPMI-FCS at 37°C before fixation and staining. Where indicated, 200 ng/ml IFN-β was added to cultures that had been previously infected for 3 h and washed for removal of extracellular parasites and cultivated for 72 h in RPMI-FCS. The number of intracellular parasites was determined under a light microscope. The experiment was repeated at least 3 independent times. The graphs show a representative experiment of the means ± SD of technical replicates in triplicate. Statistical significance was assessed using two-way ANOVA with Sidak’s multiple comparisons test. **(C)** Stationary-phase promastigotes of *L. donovani* were injected in 129Sv/Ev or *pkr* ^-/-^ mice (3 × 10^7^ parasites/animal in PBS). After 7 days, individual spleens and livers were macerated in medium, and aliquots were taken to assess parasite load by limiting dilution and calculated as parasites/organ. Graphs show the mean ± SEM of the combined datapoints from 3 independent experiments. Statistical analyses were performed using an unpaired t test. *p < 0.05, **p < 0.01.

We had previously shown that *L. donovani* requires IFN-I, more specifically, IFN-β, to thrive in macrophages of C57BL/6 mice, and that the induction of IFN-I involved NE (15). We also demonstrated that the positive influence of NE on macrophage infections requires TLR4. The expression of the NE inhibitor ISP2 in an *L. donovani* transgenic line prevented NE activation and reduced IFN-β production. Since, in *L. major* infections, we had observed that the NE-TLR4 axis leading to IFN-I was conveyed by PKR (13), we asked if this pathway was also employed by *L. donovani*. To address that, we used macrophages derived from *pkr* ^-/-^ mice, in comparison to the parental genetic background, 129Sv/Ev mice (**Figure 1**). We could readily detect the gene expression of both IFN-α (**Figure 1A**) and IFN-β (**Figure 1B**) in macrophages from 129Sv/Ev mice infected with *L. donovani* for 2 h or 6 h, in relation to control uninfected macrophages, but not in macrophages infected with *L. donovani* expressing ISP2, similarly to that which has been described for macrophages from C57BL/6 mice (15). This correlates the induction of IFN-I gene expression with the activity of serine peptidases sensitive to inhibition by ISP2, likely NE. In contrast, there was no detectable gene expression of either IFN-α (**Figure 1C**) or IFN-β (**Figure 1D**) in infected macrophages derived from *pkr* ^-/-^ mice, linking PKR to the induction of IFN-I gene expression by *L. donovani*. To evaluate further the impact of PKR on *L. donovani* infection, macrophages were infected *in vitro* and the intracellular parasite burden was estimated over 72 h (**Figure 2A**). Parasite burden increased after 48 h in 129Sv/Ev parental macrophages (**Figure 2A**, white bars), but decreased after 72 h, which agrees with the observation that those mice are resistant to some extent to *L. donovani* (17). In *pkr* ^-/-^ macrophages, we observed a reduction in parasite burden from 24 h to 72 h, as compared to the number of parasites internalized at 3 h (**Figure 2A**, dark bars), suggesting that lack of PKR contributes to parasite killing. However, numbers of intracellular parasites increased after 72 h in macrophages from both 129Sv/Ev and *pkr* ^-/-^ mice upon supplementation with exogenous IFN-β (**Figure 2B**), showing that the level of IFN-β production is key for sustained *L. donovani* infection in macrophages. The contribution of PKR to infection was further assessed in mice at 7 days post-infection (**Figure 2C**). The averages of organ masses between 129Sv/Ev and *pkr* ^-/-^ mice at 7 days post-infection were not significantly different (**Supplemental Figure 1**). We observed a significant 5-fold reduction of parasite burden in the spleens of *pkr* ^-/-^ mice, as compared to parental 129Sv/Ev mice, while the average burdens in the livers were too heterogeneous among the infected mice to allow statistical significance, albeit being, on average, 100-fold lower in *pkr* ^-/-^ mice as compared to 129Sv/Ev mice. Together, these observations support a role for PKR and IFN-β in promoting *L. donovani* infection.

**Figure 3:**
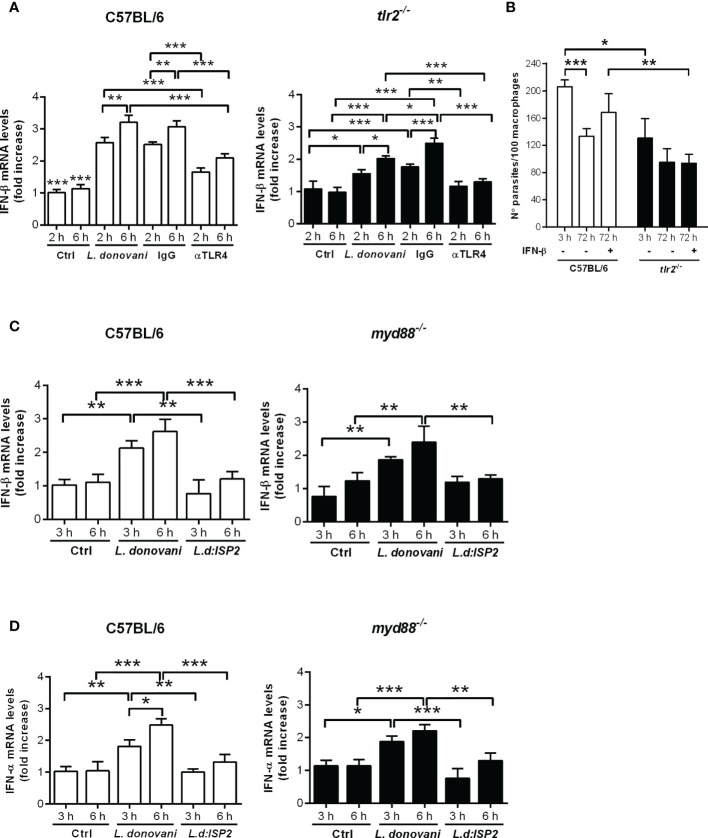
Dual role for TLR4 and TLR2 in the induction of type I IFN by *L. donovani* in macrophages. Total RNA was extracted from *L. donovani*-infected macrophages from C57BL/6, *tlr2* ^-/-^ and *myd88* ^-/-^ mice. Thioglycolate-recruited peritoneal macrophages were infected with stationary-phase promastigotes of *L. donovani* wild-type or *L. donovani* expressing ISP2 (*L.d:ISP*2) at a 10:1 parasite:macrophage ratio. After 2 or 3 h of infection, cultures were washed and RNA was extracted or cultures were incubated in RPMI-BSA for 6 h before extraction. Total RNA was extracted using the RNeasy kit (Qiagen). cDNA was made and used as templates in qPCR for the determination of the relative mRNA levels for IFN-β and IFN-α. Graphs show IFN-β levels in **(A)** C57BL/6 and *tlr2* ^-/-^ macrophages from the same experiment, and in **(C)** C57BL/6 and *myd88* ^-/-^ macrophages from the same experiment. **(D)** Graphs of the IFN-α levels in C57BL/6 and *myd88* ^-/-^ macrophages. Where indicated, cells were pre-treated with IgG or neutralizing anti-TLR4 antibodies for 30 min prior to infection. Uninfected cells were used as a control (Ctrl). The experiment was repeated 2 independent times. The graphs show representative experiments with the means ± SD of the triplicate technical replicates. Statistical significance was assessed using one-way ANOVA with Bonferroni posttest. *p < 0.05, **p < 0.01, ***p < 0.001. Differences between the same time point or the same group are shown. **(B)** Peritoneal thioglycolate-recruited macrophages from C57BL/6 and *tlr2* ^-/-^ mice were cultivated on glass coverslips overnight in RPMI-FCS and then washed and infected with late-stage promastigotes of *L. donovani* at a 5:1 parasite:macrophage ratio for 3 h at 37°C in RPMI-BSA. After 3 h, the monolayers were washed with HBSS to remove extracellular parasites, fixed with methanol and Giemsa-stained, or further incubated for 72 h in RPMI-FCS at 37°C before fixation and staining. Where indicated, 200 ng/ml IFN-β was added to cultures that had been previously infected for 3 h and washed for removal of extracellular parasites and cultivated for 72 h in RPMI-FCS. The number of intracellular parasites was determined under a light microscope. The experiment was performed 3 independent times. The graphs show a representative experiment of the means ± SD of replicates in triplicate. Statistical significance was assessed using two-way ANOVA with Tukey’s and Sidak’s multiple comparison tests. *p < 0.05, **p < 0.01, ***p < 0.001.

**Figure 4:**
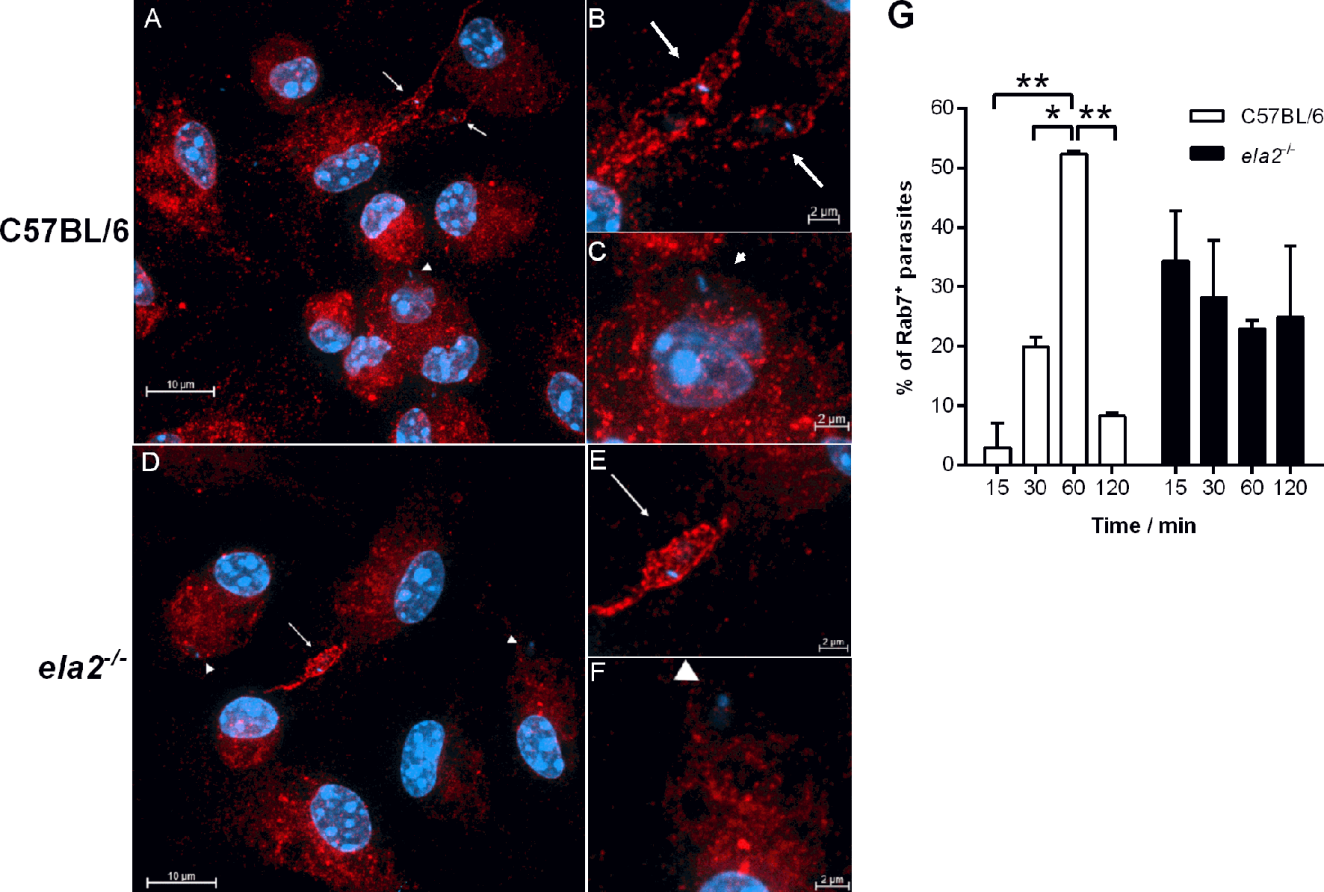
Neutrophil elastase influences the kinetics of acquisition of endosomal markers in parasite-containing phagosomes. Peritoneal thioglycolate-recruited macrophages from C57BL/6 and *ela2^-/-^* mice were cultivated on glass coverslips overnight in RPMI-FCS and then washed and infected with late-stage promastigotes of *L. donovani* at a 20:1 parasite:macrophage ratio for 15 min at 37°C in RPMI-BSA. After, the monolayers were extensively washed with HBSS to remove extracellular parasites, fixed with paraformaldehyde, or chased for up to 2 h in RPMI-FCS at 37°C, before fixation at the time points indicated in the figure. Cells were labeled with anti-Rab7 antibodies, followed by the appropriate secondary antibody conjugated to Cy3. **(A)** C57BL/6 macrophages were infected and chased for 60 min. Arrows point to Rab7^+^ co-localized parasites and arrowheads point to Rab7^-^ non-co-localized parasites with the respective magnified images in **(B, C)**; **(D)** *ela2^-/-^* macrophages infected and chased for 60 min, **(E, F)** with magnified images. Images were acquired on a confocal microscope in 0.48-μm slices from bottom to top, and images show all slices stacked in the maximum intensity projection (MIP). **(G)** Quantification of the proportion of Rab7^+^ parasite-containing compartments at the different time points. Graphs show means ± SD of replicates. Statistical significance was assessed using two-way ANOVA with Tukey’s and Sidak’s multiple comparison tests. *p < 0.05, **p < 0.01.

We had demonstrated that the production of IFN-I by infected macrophages is dependent on NE, influencing parasite burdens in macrophages, and that TLR2 and TLR4 were required for optimal parasite intracellular development (15). Next, we asked if those TLRs participate in the induction of IFN-β gene expression. To that end, we used neutralizing antibodies to block TLR4 in C57BL/6 macrophages prior to infection (**Figure 3A**). Upon infection, the gene expression of IFN-β was increased nearly 3-fold in infected untreated macrophages or in macrophages pre-incubated with control IgG, as compared to uninfected controls, while the gene expression of IFN-β increased by 1.5-fold in C57BL/6 macrophages pre-treated with anti-TLR4 antibodies, denoting 50% less induction of IFN-β gene expression as compared to untreated macrophages (**Figure 3A**, left). Likewise, we observed nearly 1.5-fold increased gene expression of IFN-β upon infection of macrophages from *tlr2* ^-/-^ mice (**Figure 3A**, right), as compared to uninfected macrophages, suggesting also partial contribution of TLR2 for IFN-β induction. Finally, we verified if both TLRs are simultaneously required to induce optimal induction of IFN-β gene expression by blocking TLR4 in *tlr2* ^-/-^ macrophages with neutralizing antibodies. Pre-treatment with anti-TLR4, but not with control IgG, completely abolished induction of IFN-β gene expression in infected *tlr2* ^-/-^ macrophages, indicating the dual contribution of TLR2 and TLR4 for the induction of optimal IFN-β gene expression, which should impact parasite intracellular development. However, while exogenous IFN-β improved intracellular parasite burdens after 72 h in macrophages from C57BL/6 mice, it was unable to recover intracellular parasite growth/development in the absence of TLR2 (**Figure 3B**). This observation shows that although TLR2 can contribute to the induction of IFN-β gene expression, lack of sufficient IFN-β alone cannot account for the impaired parasite development in *tlr2* ^-/-^ macrophages. Indeed, it was shown that TLR2 is involved in the parasite-induced modulation of phagosome maturation shortly after phagocytosis (7), which is likely not influenced by IFN-I. Finally, the participation of the adaptor protein MyD88 downstream of TLRs in the induction of IFN-I was ruled out, as its absence in MyD88 knock-out macrophages did not affect the increased gene expression of IFN-β (**Figure 3C**) or of IFN-α (**Figure 3D**) upon infection with *L. donovani.* This is in line with the current knowledge that IFN-related responses downstream of TLRs occur mainly *via* the TRIFF adaptor protein (18). There was no increase in IFN-I in macrophages infected with the *L. donovani* transgenic line expressing ISP2 (*Ld:ISP*2), confirming our previous observation for the requirement of serine peptidase activity to engage TLR4-dependent induction of IFNs-I expression (15). In line with a crucial role for the modulation of phagosome maturation *via* fusion with compartments of the endosomal system for *L. donovani* survival, we evaluated the kinetics of acquisition of the late endosome marker Rab7 in compartments harboring parasites after parasite uptake (**Figure 4**). This approach provides a read-out for assessing the potential influence of NE in the kinetics of vesicle fusion after infection. Although Rab7 labeling was intense and distributed throughout the cells, Rab7^+^ parasites could be confidently discriminated by observing the morphology of the entire promastigote cell body including the flagellum, labeled for Rab7, likely due to close juxtaposition between the phagosome and the parasite membranes (**Figures 4A, B**, arrows). Rab7^-^ parasites were identified by observing DAPI-stained kinetoplasts inside macrophages, but devoid of red fluorescence shaping of the parasite membranes (**Figures 4A, C**, arrowheads). Rab7^+^ parasites (**Figures 4D, E**) and Rab7^-^ parasites (**Figures 4D, F**, arrowheads) were also observed in macrophages from *ela2* ^-/-^ mice. The quantification of the proportion of Rab7^+^ parasites at different time points after interaction with macrophages revealed that, in macrophages from C57BL/6 mice, the acquisition of this marker peaked at 60 min after internalization, and reduced at 120 min, likely reflecting phagosome maturation moving forward with time (**Figure 4G**, white bars). In contrast, we did not observe this well-defined pattern of acquisition of Rab7 in *L. donovani*-containing compartments in macrophages from *ela2* ^-/-^ mice (**Figure 4G**, dark bars). Those observations suggest that NE not only induces late responses related to gene expression and parasite survival, such as expression of IFN-I, but also participates in crucial steps of the host cell–parasite interface at early times. This is consistent with our observations that NE and TLR4 co-localize with *L. donovani* at the point of entry in macrophages and remain co-localized in mature parasitophorous vacuoles 72 h after infection (15).

**Figure 5:**
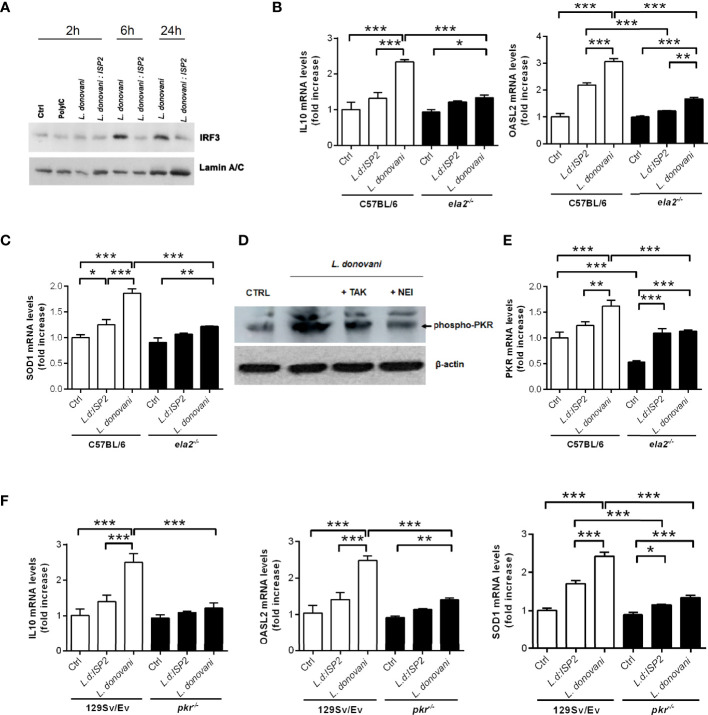
Neutrophil elastase and PKR are required for the induction of anti-inflammatory genes in infected macrophages. **(A)** Nuclear extracts were obtained from C57BL6 macrophages infected for 2, 6, or 24 h with stationary-phase promastigotes of *L. donovani* at a 10:1 parasite:macrophage ratio. The nuclear extracts were processed for Western blot analysis with antibodies to IRF3, as well as nuclear lamin for the loading control. Macrophages incubated in medium alone were used as the baseline control (Ctrl). Poly I:C was used at 25 µg/ml. In **(D)**, total lysates were obtained from C57BL/6 macrophages infected for 1 h with stationary-phase promastigotes of *L. donovani* at a 10:1 parasite:macrophage ratio and processed for Western blot with antibodies to phospho-PKR, as well as actin for the loading control. Where indicated, monolayers were pre-treated with 100 nM of the TLR4 inhibitor, TAK-242 or with 10 µM of neutrophil elastase inhibitor (NEI) before infection. Total RNA was extracted from *L. donovani*-infected macrophages from **(B, C, E)** C57BL/6 and *ela2* ^-/-^ mice or **(F)** 129Sv/Ev and *pkr* ^-/-^ mice. Thioglycolate-recruited peritoneal macrophages were infected with stationary-phase promastigotes of *L. donovani* wild-type or *L. donovani* expressing ISP2 (*L.d:ISP*2) at a 10:1 parasite:macrophage ratio for 2 h, cultures were washed, and RNA was extracted or cultures were incubated in RPMI-BSA until 6 h before extraction. cDNA was made and used as a template in qPCR for the determination of the relative mRNA levels for IL10, OASL2, SOD1, and PKR as indicated. The experiment was repeated 2 independent times. The graphs show representative experiments, with the means ± SD of the triplicate technical replicates. Statistical significance was assessed using one-way ANOVA with Bonferroni posttest. *p < 0.05, **p < 0.01, ***p < 0.001.

It was previously shown that in macrophage infections with *L. amazonensis*, not only is PKR activated, but its expression is also significantly increased, creating a positive feedback loop resulting in high levels of both IFN-β and IL10 (9). In an interesting parallel, the infection of macrophages by another phagosome-resident microbe, *Mycobacterium leprae*, also induces classical anti-viral responses including IFN-β expression *via* interferon regulatory factor 3 (IRF3) and downstream genes, such as 2′-5′-oligoadenylate synthetase (OAS)-like (OASL2), that allow microbial survival and growth (19). Next, we asked if the NE-dependent route triggered by *L. donovani* affects the expression of additional genes related to IFN-I. At 2 h post-infection, we did not observe changes in the nuclear accumulation of IRF3, as compared to control macrophages or to macrophages treated with the synthetic double-stranded RNA Poly:IC. Importantly, we observed that while *L. donovani* induces the accumulation of IRF3 in the nuclear fraction of macrophages at 6 h and 24 h post-infection, this does not occur upon infection with *L. donovani*:ISP2, linking a serine peptidase activity-dependent pathway to IRF3-related IFN-I response (**Figure 5A**). Considering that we have previously demonstrated that ISP2 targets mainly NE activity at the surface of macrophages (12), it is expected that the inability of *L. donovani*:ISP2 to induce accumulation of nuclear IRF3 results from the inhibition of the NE-TLR4 pathway. Furthermore, C57BL/6 macrophages infected with *L. donovani*, but not macrophages from *ela2* ^-/-^ mice, exhibited significantly increased expression of IL10, OASL2 (**Figure 5B**), and superoxide dismutase (SOD1) genes (**Figure 5C**). Parasites expressing ISP2 did not induce significant expression of the tested genes, as compared to control, providing additional support for a role of NE activity in the induction of IL10, SOD, and PKR in macrophages from C57BL/6 mice. Those results show that the parasite uses the NE-TLR4 pathway to induce a gene expression pattern compatible with anti-inflammatory stimuli and pro-microbial growth. Similarly to that described for macrophages infected with *L. amazonensis* (9), we detected the phosphorylation of PKR upon infection with *L. donovani*, directly showing that PKR activation occurs in response to infection (**Figure 5D**). Parasite-induced phosphorylation was prevented by macrophage treatment with an inhibitor of TLR4 signaling (TAK) (20), or with an irreversible inhibitor of NE activity, placing PKR activation as a downstream event of TLR4 activation. The expression of the PKR gene increased in comparison to uninfected controls (**Figure 5E**), showing similarity to that observed in *L. amazonensis* infection (9). The participation of PKR in the pro-microbial pattern of gene expression was further analyzed. We observed again the induction of IL10, OASL2, and SOD1 gene expression in infected macrophages from 129Sv/Ev mice (**Figure 5F**), but not in *pkr* ^-/-^ macrophages, establishing a connection between the NE-TLR pathway converging to responses conveyed by PKR to induce the expression of IFN-I and anti-inflammatory genes, and promote parasite development in macrophages.

**Figure 6:**
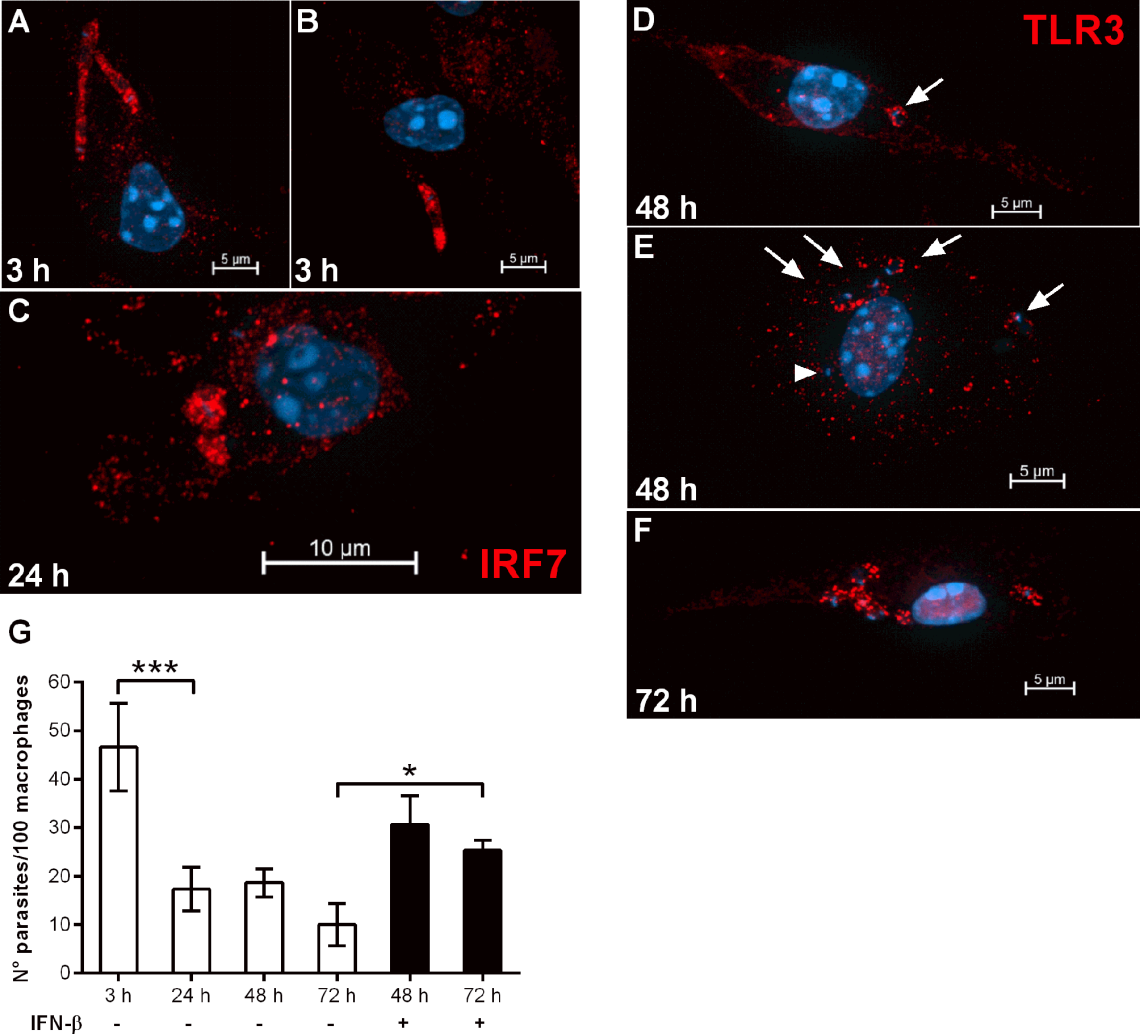
IRF7 and TLR3 are recruited to *L. donovani* phagosomes and IFN-β can revert macrophage leishmanicidal activity. **(A–F)** Thioglycolate-recruited macrophages from C57BL/6 mice were cultivated on glass coverslips overnight in RPMI-FCS and then washed and infected with late-stage promastigotes of *L. donovani* at a 10:1 parasite:macrophage ratio for 3 h at 37°C in RPMI-BSA. After, the monolayers were extensively washed with HBSS to remove extracellular parasites, fixed with paraformaldehyde or cultivated up to 72 h (as indicated in the images) in RPMI-FCS at 37°C, before fixation. Cells were labeled with **(A–C)** anti-IRF7 antibodies or **(D–F)** anti-TLR3 antibodies, followed by the appropriate secondary antibody conjugated to Cy3. Arrows point to TLR3 co-localized with parasites and arrowheads point to parasites not co-localized with TLR3. **(G)** Macrophages of the 14M1.4 cell line were treated with mitomycin C, washed, and infected with late-stage promastigotes of *L. donovani* at a 5:1 parasite:macrophage ratio in DMEM-BSA. After 3 h, the monolayers were washed with HBSS to remove extracellular parasites, fixed with paraformaldehyde and Giemsa-stained, or further incubated for 24 h, 48 h, or 72 h in DMEM-FCS at 37°C before fixation and staining. Where indicated, 200 ng/ml IFN-β was added to cultures that had been previously infected for 3 h and washed for removal of extracellular parasites, and cultivated for 48 h or 72 h in DMEM-FCS. The number of intracellular parasites was determined under a light microscope. Statistical significance was assessed using one-way ANOVA with Bonferroni posttest. *p < 0.05, ***p < 0.001.

Previous work with a different *L. donovani* strain (LV9) has reported that parasites were rapidly killed by immortalized bone marrow stromal macrophages *in vitro* by temporally spaced mechanistic events relying on nitric oxide (up to 12 h) and on the central regulator of type I interferon response, IRF7 (12-48 h) (16). In this model, parasites induced IRF7 expression, and its recruitment to parasite-containing phagosomes as early as 6 h, with continuous increase in the accumulation of IRF7 in the parasitophorous vacuole up to 48 h (16). Likewise, we promptly detected co-localization between IRF7 and *L. donovani*-containing phagosomes at 3 h post-infection in C57BL/6 macrophages (**Figures 6A, B**), and its accumulation in amastigote-containing compartments at 24 h (**Figure 6C**). Even though Phillips and co-authors were able to determine that IRF7 was intra-phagosomal and closely juxtaposed to amastigotes (16), we were unable to confidently discriminate if IRF7 was intra-phagosomal. TLR3 was also found in *Leishmania*-containing phagosomes at different time points of infection (**Figures 6D**–**F**). While at 48 h, most parasitophorous vacuoles were TLR3^+^ (**Figures 6D, E**, arrows), we also observed a few parasites that did not co-localize with TLR3 (**Figure 6E**, arrowhead). At 72 h, all parasite-containing compartments were TLR3^+^, suggesting that the recruitment of TLR3 to phagosomes might be a continuous event that peaks at latter times of intracellular amastigote growth. IRF7 was linked to the killing of *L. donovani* (LV9) in a way partially, but not entirely, dependent of IFNα. We then evaluated the response of the stromal macrophage cell line 14M1.4 to infection with the *L. donovani* MW897 strain used throughout our study, testing the capacity of *L. donovani* to establish infection in those macrophages by following parasite loads at different time points (**Figure 6G**). As shown for *L. donovani* LV9, we found that *L. donovani* MW897 is likewise efficiently killed by those macrophages *in vitro*. However, the addition of exogenous IFN-β recovered sustained infection at 72 h, denoting that the requirement of IFN-β for successful infection is a common trend in macrophage populations of different origins.

**Figure 7:**
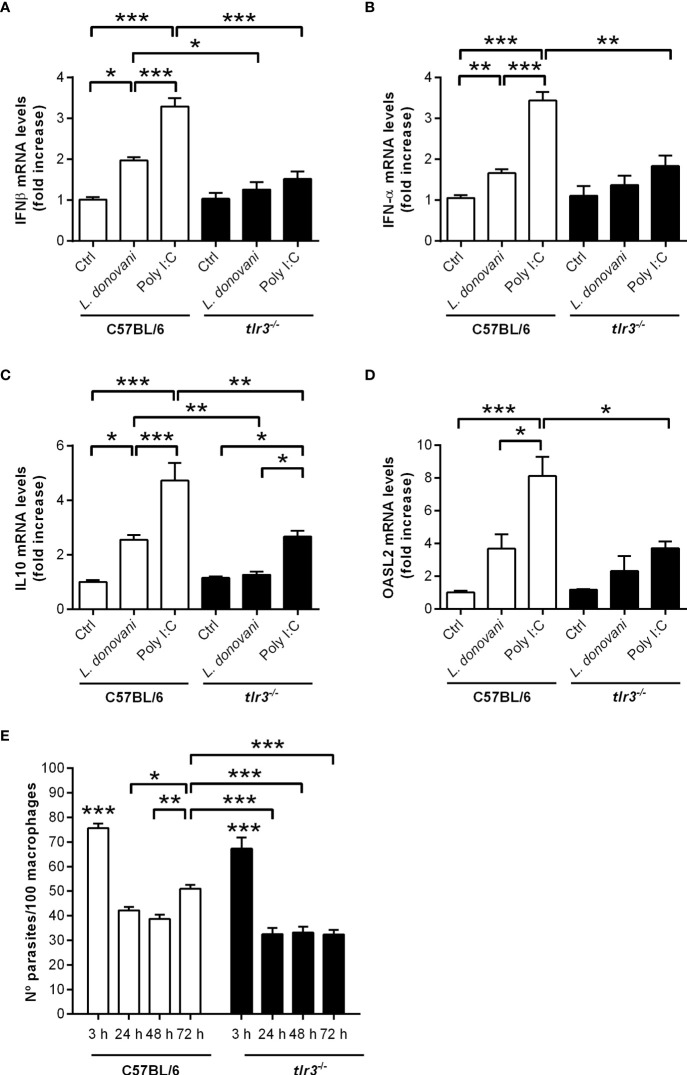
TLR3 is required for the induction of type I IFN, IL10, and OASL2 and for parasite growth in macrophages. Thioglycolate-recruited peritoneal macrophages were infected with stationary-phase promastigotes of *L. donovani* at a 10:1 parasite:macrophage ratio for 2 h, cultures were washed, and RNA was extracted or cultures were incubated in RPMI-BSA until 6 h before extraction. cDNA was made and used as templates in qPCR for the determination of the relative mRNA levels for **(A)** IFN-β, **(B)** IFN-α, **(C)** IL10, and **(D)** OASL2. Poly I:C was used at 25 µg/ml. The experiment was repeated 2 independent times. The graphs show representative experiments, with the means ± SD of the triplicate technical replicates. Statistical significance was assessed using one-way ANOVA with Bonferroni posttest. **(E)** Peritoneal thioglycolate-recruited macrophages from C57BL/6 and *tlr3* ^-/-^ mice were cultivated on glass coverslips overnight in RPMI-FCS and then washed and infected with late-stage promastigotes of *L. donovani* at a 5:1 parasite:macrophage ratio for 3 h at 37°C in RPMI-BSA. After 3 h, the monolayers were washed with HBSS to remove extracellular parasites, fixed with methanol and Giemsa-stained, or further incubated for 24 h, 48 h, or 72 h in RPMI-FCS at 37°C before fixation and staining. The number of intracellular parasites was determined under a light microscope. The experiment was performed 2 independent times. The graphs show a representative experiment of the means ± SD of technical replicates in triplicate. Statistical significance was assessed using two-way ANOVA with Tukey’s and Sidak’s multiple comparison tests. *p < 0.05, **p < 0.01, ***p < 0.001.

Finally, endosomal TLR3 is known to act upstream of IRF3 activation, leading to proinflammatory cytokines, IFN-I, and IFN-responsive genes (21–23). We asked if TLR3 participates in the IFN-I response induced by *L. donovani* in macrophages, with consequences to parasite long-term intracellular growth. First, the potential presence of the *Leishmania* endogenous RNA-virus (LRV) was discarded (**Supplementary Figure 2**). We did not detect increased gene expression of either IFN-β (**Figure 7A**) or IFN-α (**Figure 7B**) in macrophages from TLR3 knock-out mice, indicating the requirement of TLR3 for the induction of IFN-I by *L. donovani*. Furthermore, the induction of IL10 and of SOD1 gene expression was also dependent on TLR3 (**Figures 7C, D**), showing that the anti-viral-type responses conveyed *via* PKR and TLR3 are mobilized by *L. donovani* to downmodulate the inflammatory response of macrophages, likely influencing infection. In agreement with that, there was no increase in the numbers of intracellular parasites at 72 h in *tlr3* ^-/-^ macrophages (**Figure 7E**), demonstrating that this endosomal receptor also contributes to the survival/development of *L. donovani* in macrophages. As expected, macrophages from TLR3 knock-out mice did not induce expression of either IFNs-I in response to the synthetic double-stranded RNA Poly I:C, but some degree of IL10 gene expression was detected. The residual induction of IL10 by Poly:IC in those macrophages, although much lower than that in macrophages from C57BL/6, could result from engagement of PKR, for example, leading to some induction of IL10 that is independent of TLR3.

**Figure 8:**
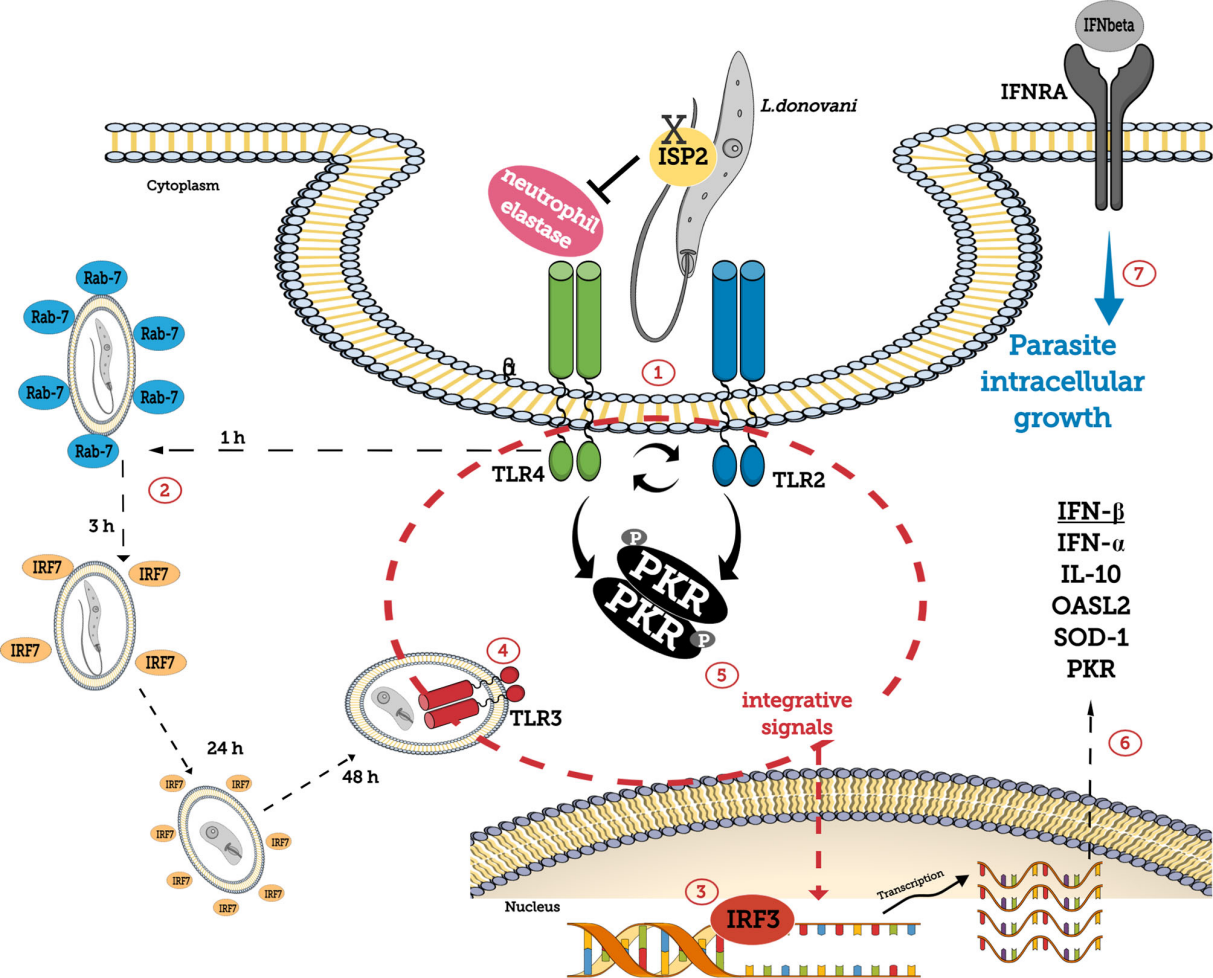
Schematic representation of the role of TLRs and PKR in *L. donovani* development in macrophages. *L. donovani* utilizes macrophage NE activity, susceptible to inhibition by the ecotin-like inhibitor of serine peptidase ISP2, *via* TLR4 and TLR2 during phagocytosis (1), to induce PKR phosphorylation. NE contributes to the modulation of Rab7^+^ endosomal fusion with the phagosome (2) and to IRF3 accumulation in the nucleus (3). IRF7 recruitment to parasite-containing phagosomes takes place during early stages of infection (2). TLR3 is recruited to the parasitophorous vacuole (4), and together with integrative signals conveyed through PKR (5) acts as a hub to induce the expression of IFN-I, OASL2, SOD1, IL10, and PKR genes (6). IFNβ ensures parasite survival and growth in macrophages (7).

In summary, we provide evidence that *L. donovani* engages NE-TLR4 and TLR2 during phagocytosis, which contribute to parasite fate through modulation of endosomal fusion with the phagosome, and in combination with TLR3 induces classical anti-viral type innate responses conveyed by PKR, leading to the expression of IFN-I, OASL2, SOD1, and IL10 genes and promoting sustained parasite burdens in macrophages (**Figure 8**).

## Discussion

Although experimental murine VL differs from human disease in several aspects, it shares severe splenomegaly and tissue remodeling, making it a model for the understanding of molecular mechanisms of the disease (24). The onset of disease results from a complex network of interactions that include loss of certain cellular sub-types, vascular remodeling, and a delicate balance of cytokine production, making it challenging, nevertheless relevant, to address specific mechanistic pathways in infected cells. In mice, *L. donovani* has been shown to reside in a multiplicity of myeloid cellular sub-populations, including bone marrow cells, newly recruited monocytes to the spleen, macrophages of the red pulp, marginal zone macrophages (MZM), and marginal metallophilic macrophages (MMM), as well as in liver Kupffer cells (6). However, due to the complexity of performing *in vitro* studies with Kupffer cells or splenic macrophages, the identification of molecular pathways exploited by *L. donovani* to survive in macrophages have been addressed using other more laboratory-amenable macrophage populations (16, 25). Here, we used murine peritoneal macrophages to address innate molecular pathways triggered by *L. donovani*.

We had previously shown that NE and TLR4, at the surface of macrophages, are engaged by the parasite, and required for sustained macrophage infection (15). Although IFN-α was shown to be deleterious for *L. donovani* in other macrophage infection models (16), we showed previously that it did not influence the ability of *L. donovani* to grow in murine peritoneal macrophages *in vitro* (15). On the other hand, IFN-β is required, at least so in macrophages from C57BL/6 mice, and we confirmed here that this is also the case for macrophages from 129Sv/Ev mice. More recently, both IFN-α and IFN-β were found as the major regulators upstream of CD4 T cell responses in VL patients, suppressing IFN-γ production, and mice defective in IFN-I showed improved control of parasite burdens (26). Although the cellular sources of IFN-I in infected mice were not determined, this connects our findings of IFN-I gene expression by the infected macrophage with an important step for host susceptibility to infection and to splenic pathology. Even though IFN-I responses could be triggered simultaneously by diverse cell types in an inflammatory environment, our findings suggest that infected macrophages could contribute to this scenario. Furthermore, our observations suggest that the threshold levels of IFN-β are crucial for the parasite’s intracellular fate, since we could restore parasite burdens in macrophages of 129Sv/Ev mice by addition of exogenous IFN-β. The beneficial effect of IFN-β to sustain parasite burden was likewise observed when immortalized bone marrow stromal macrophages (14M1.4) were used. Importantly, this cell line was previously shown to efficiently kill intracellular *L. donovani* (LV9 strain) by mechanisms related to IFN-α and dependent on IRF7 (16), a key element in the amplification of type I interferon pathways. The authors found that recruitment of IRF7 to phagosomes was specific to infection with *Leishmania*, as it did not occur when latex beads were internalized by macrophages that had been pre-treated with Poly I:C. We also found recruitment of IRF7 to parasite-containing phagosomes early on in infected macrophages of C57BL/6 mice, which was maintained at 24 h, showing similarities between the infection of those two *L. donovani* strains, LV9 and MW897, in different macrophage populations. The capacity of IFN-β to revert parasite killing in this cell type highlights the central role of IFN-β for *L. donovani* successful infection. It remains to be tested if this assumption holds for parasite intracellular development in a wide range of different macrophage sub-populations. Noteworthy, splenic MZMs and MMMs were described as the major cellular sources of IFN-I in an infection model with herpes simplex (27), showing that the cellular sub-sets that are hosts for *L. donovani* in the spleen can produce high levels of IFN-β as a result of anti-viral responses.

We found that *L. donovani* uses both TLR4 and TLR2 in an additive way to induce IFN-I gene expression, since blockade of TLR4 or lack of TLR2 led to half the gene expression of IFN-β, as compared to untreated infected macrophages. Blocking TLR4 in *tlr2* ^-/-^ macrophages finally prevented expression of the IFN-β gene, denoting that both receptors act independently to induce IFN-I gene expression in infected macrophages. We have previously reported that the induction of IFN-β *via* TLR4 by *L. major* required NE activity (13) and that *L. donovani* also needs NE to promote IFN-I gene expression (15). Here, we connected TLR4 to *L. donovani* induction of IFN-I, together with NE. It is feasible that the levels of TLR expression in macrophage sub-populations in the spleen, together with additional sources of NE, such as infiltrating neutrophils, can correlate with more efficient IFN-I response. On the other hand, *L. donovani* isolates potentially expressing high levels of ISP2, thus prone to inactivate NE, could have a disadvantage in sustaining chronic infection. It makes sense that TLR4 and TLR2 act separately, in view of previous findings that parasite cell surface LPG, a major TLR2 ligand, led to IFN-I expression in macrophages infected with *L. amazonensis* (10). Although not explored here, it is feasible that *L. donovani* LPG also plays a role in the induction of IFN-I *via* TLR2 in our model, providing an explanation for the dual role of those cell surface TLRs. LPG is known to play a role at the initial stages of parasite uptake, since phagosome maturation occurs faster in macrophages infected with *lpg* ^-/-^ mutants (28). It was also observed that *L. donovani* was unable to delay phagosome maturation in *tlr2* ^-/-^ macrophages, leading to the proposal that TLR2 is a key element to control the kinetics of phagosome maturation and the formation of a parasitophorous vacuole permissive for parasite growth (7). Intriguingly, we have observed that NE also seems to play a role in the kinetics of acquisition of endosomal compartments in *L. donovani* phagosomes. In the absence of NE, we found similar percentages of parasite-containing phagosomes positive for Rab7 at different time points during the kinetics, suggesting a deregulation of the timely events leading to vesicle fusion to *Leishmania*-containing phagosomes, and potentially of phagosome maturation. In addition, NE was also important for the induction of SOD1 gene expression, presumably aiding in the detoxification of superoxide anions and contributing to parasite survival. At present, we do not know how NE participates in this process; however, its association with TLR4 might offer an explanation. Although TLR4 was shown to accelerate the maturation of bacterium-containing phagosomes (29), experiments of phagosome maturation after particle uptake discarded the participation of those receptors, at least so for the maturation of phagosomes containing inert particles bearing TLR ligands (30). Years later, TLR4 was implicated in restraining lysosome fusion to phagosomes in dendritic cells, leading to cross-presentation (31). Although it is still controversial that TLR4 might influence phagosome maturation, depending on the ligand–host cell combination model, the NE-TLR4 platform could potentially play a role in the kinetics of vesicle fusion in *L. donovani*-infected macrophages, as shown for TLR2 (7). The induction of IFNs-I is known to be conveyed by endosomal TLRs, and endosomal TLR4 induces IFN-I *via* TRIF and TRAM (18). Endosomal TLR2 (heterodimer TLR1/TLR2) can also induce IFN-I (32), *via* TRAM, but MyD88 is involved (33), and we have discounted the requirement of MyD88 for the induction of IFN-I gene expression. Currently, we do not know the mechanism by which TLR2 contributes to the induction of IFN-I, but it is possible that it involves its interaction with additional, yet unidentified, receptors. Indeed, TLRs are known to cross-talk with numerous receptors, including G-coupled protein receptors (GPCRs), complement receptors (34), and low-density lipoprotein-related receptor (LRP1) (35), engaging in downstream pathways that include protein kinase activation, recruitment of Rabs, and the regulation of responses from endocytic membranes. Interestingly, the TLR-LRP1 crosstalk in murine macrophages leads to cell reprogramming and to the suppression of inflammatory responses. We have shown the co-location between TLR4-NE (15) and between CD11b-NE (12) in macrophages. It is possible that signaling platforms bearing TLR4-NE-CD11b and, in parallel, bearing TLR2 and additional receptors are formed at the cell surface upon parasite binding and phagocytosis, influencing the kinetics of parasite uptake and/or how downstream signals take place thereafter from the phagosome. In this scenario, IFN-I induction could result independently from signals triggered by the TLR4-NE-CD11b platform *via* TRIF, and by receptors associated with TLR2, and lack of MyD88 should not influence such interactions or the induction of IFN-I gene expression. We found increased nuclear IRF3 in infected macrophages, which fits well with a signaling pathway downstream of TLR4-TRIF. Considering that *L. donovani*:*ISP*2 did not induce enhanced IRF3, it is plausible to connect the NE-TLR4 pathway to the induction of IRF3 and, ultimately, to IFN-I gene expression. The induction of IFN-β *via* TLR4 also involves IRF7 *via* TRIF, but not MyD88 (36), and we found recruitment of IRF7 to the phagosome in infected macrophages, but we do not know if or how this affects IFN-I. Nuclear IRF3 is likewise a result of TLR3 activation with signaling through TRIF (18), and we detected TLR3 in the phagosome at 48–72 h. Those findings connect several pathways related to endosomal TLRs and TRIF signaling to favor *L. donovani* development in macrophages. All those players might contribute to some extent to the induction of IFN-I, thus, when this intricate network is affected, it results in less-than-optimal IFN-I induction.

We found that the upstream signals leading to increased IFN-I gene expression in the infected macrophage converge to PKR, which proved to be essential for IFN-I induction and for parasite intracellular development. We also detected PKR phosphorylation upon infection in a TLR4-dependent and NE-dependent manner, undoubtfully linking TLR4 to PKR activation in the context of macrophage infection by *L. donovani*. In addition, we found that PKR is also at the center of the induction of other genes, such as IL10, SOD1, and OASL2. SOD1 and IL10 provide the means to attenuate macrophage microbicidal responses. In a broader context, it was more recently described that in *Leishmania-*infected macrophages, the induction of the antioxidant response *via* the transcription factor Nuclear Factor Erythroid 2-Related Factor 2 (NFR2), which acts downstream of PKR (37), reprograms host cell metabolism and promotes parasite survival (38). IL10 has been extensively documented for its suppressive role in VL (39, 40). Considering that, in our model, we do not use bone marrow naïve macrophages, but thioglycolate-recruited macrophages that were primed *in vivo* for an M1 phenotype, the induction of anti-inflammatory and pro-microbial genes upon infection illustrates the capacity of *L. donovani* to steer the effector function of an inflammatory-committed macrophage in its favor, at least so, *in vitro*. Also, it provides evidence that the parasite hijacks receptors classically involved in anti-microbial responses, such as TLRs, PKR, and NE to induce molecules capable of attenuating the microbicidal environment. Importantly, *pkr* ^-/-^ mice had significantly reduced parasite burdens in the spleen, which fits well with similar findings for mice lacking the IFN-α/β receptor (*ifnar^-/-^*) (26). This suggests that PKR is relevant to foster infection *in vivo*, likely because it is a key element to promote IFN-I. A role for PKR in acute parasitism was more prominent in the spleen than in the liver, where we observed high burdens in both wild-type and *PKR* knock-out mice, even though average burdens in the livers of *PKR* knock-out mice were nearly 1,000-fold lower than in WT mice. Considering that murine Kupffer cells were described as defective in the production of leishmanicidal effector molecules such as ROS (41), it is possible that the induction of further immunosuppressive molecules would favor high liver parasitism during acute infection, before full anti-parasite adaptive immunity is mounted. In fact, endogenous IFNs-I are commonly present in hepatopathic livers and Kupffer cells are particularly responsive to IFNs-I (42), by producing IL10 and inhibiting IL1 production and inflammasome activation (43), which could make the liver further permissive to parasitism during early infection. At present, we do not know if those mechanisms are shared by *L. infantum* in infections in the mouse model, or in primary macrophages, and the potential role of the TLR-NE-PKR-IFN-I axis is currently under investigation. Nevertheless, in line with the participation of type I interferons in infections with *L. infantum*, the analysis of the transcriptional profile of VL patients, combined with the mouse model, provided evidence for a TLR4-IFN-β pathway in the induction of asymptomatic disease. In this study, TLR4 and IFN-β were found to dampen the Th1 inflammatory response, aiding in *L. infantum* survival while reducing chronic inflammation and pathology (44).

PKR is classically a cytoplasmic double-stranded RNA sensor in anti-viral responses. Viruses, and the consequent downstream responses, have been closely associated to severe disease in *Leishmania* infections either due to the presence of *Leishmania* viruses, mainly in *L. braziliensis* and *L. guyanensis*, or to co-infection with exogenous viruses (45, 46). However, in the case of *L. amazonensis* and PKR activation, no endogenous viruses were detected (10) and we did not detect endogenous virus in *L. donovani* either. We did not address how *L. donovani* engages PKR downstream of TLR4. More recently, a virus endogenous to the Amazonian sandfly, a phlebovirus, was found to promote *L. amazonensis* infection through the engagement of PKR-IFN-I and IL10 (47). This helps to establish a common mechanistic behavior in macrophages infected with *L. amazonensis* or *L. donovani*, whereby classical anti-viral type responses *via* IFN-I exert a pro-parasite survival role for successful infection. Likewise, the endosomal double-stranded RNA sensor TLR3 was associated with the anti-viral type response to *Leishmania* harboring endogenous viruses (48), with consequences to IFN-I production. We demonstrated that TLR3 also plays a role in the induction of IFN-I by *L. donovani* and is required for parasite growth in macrophages. Either way, both double-stranded-RNA sensors, PKR (cytoplasmic sensor) and TLR3 (endosomal sensor), are promptly engaged by *L. donovani* for survival and growth, and alternative mechanisms of activation independent of nucleic acids are currently under investigation. It was reported that the transfection of *L. donovani* DNA into macrophages induced high expression of IFN-β through a TBAK-1 and IRF3-dependent pathway, but in a TLR-independent way (49). This differs from our findings, which establish a connection between TLR4 and TLR3 to IFN-β expression at the RNA level. Although we cannot rule out that some degree of parasite killing occurs, releasing DNA into infected cells, we have unequivocally shown that the RNA sensors PKR and TLR3 are central players in the induction of IFN-β in our model. Importantly, PKR was required for the expression of additional genes that contribute to parasite survival. In particular, IL10 is known as a major player in VL, not only for ensuring macrophages as an initial replicative niche and immunomodulators (6), but also for promoting spatial segregation between dendritic cells and T cells in infected mice, causing impaired immune response and continuation of splenic pathology (50). In this context, our findings that the NE-TLR4-PKR axis is required for IL10 production by infected macrophages connects this molecular route to a central mechanism in visceral disease.

In conclusion, we have established a connection between TLR4-NE, PKR activation, and gene expression of IFNs-I, IL10, SOD-1, and OASL2 upon infection of murine macrophages by *L. donovani*. Importantly, we confirmed that full peptidase activity was required, since parasites constitutively expressing the natural serine peptidase inhibitor ISP2 were incapable of triggering such responses. TLR2 was found to contribute to the induction of IFNs-I in an additive way to TLR4, while TLR3, which was found in the parasitophorous vacuole, was found to be essential for parasite-induced IFNs-I and additional genes that can favor parasite survival. Altogether, these results help to understand how host cell surface receptors internalized with the parasite, combined with the recruitment of intracellular receptors to the phagosome, enable this pathogen to take control of the host cell innate responses to its advantage.

## Data Availability Statement

The raw data supporting the conclusions of this article will be made available by the authors, without undue reservation.

## Ethics Statement

The animal study was reviewed and approved by Comissão de Ética no Uso de Animais (CEUA) UFRJ.

## Author Contributions

AL, JM, and UL conceptualized the work and supervised experimental work. AL and JM funded the work. BD, AG, AV and TC performed experiments and analyzed data. All authors contributed to the discussion of results. AL and AG wrote the manuscript, with revisions and contributions of all authors. All authors contributed to the article and approved the submitted version.

## Funding

This work was supported by funds of CNPq grant number 311208/2017-7 (AL) (https://www.gov.br/cnpq/pt-br), FAPERJ grant number E26/202.655/2019 (AL) (http://www.faperj.br/), the Medical Research Council (MRC; MR/K019384, JM), the Newton Fund (MR/N017269/1, AL and JM) from United Kingdom Research and Innovation (UKRI) (https://www.ukri.org/), *via* the Global Challenges Research Fund under grant agreement “A Global Network for Neglected Tropical Diseases”, and Medical Research Council (MR/P027989/1, AL and JM). AL and UL are CNPq fellows. The funders had no role in study design, data collection and analysis, decision to publish, or preparation of the manuscript.

## Conflict of Interest

The authors declare that the research was conducted in the absence of any commercial or financial relationships that could be construed as a potential conflict of interest.

## Publisher’s Note

All claims expressed in this article are solely those of the authors and do not necessarily represent those of their affiliated organizations, or those of the publisher, the editors and the reviewers. Any product that may be evaluated in this article, or claim that may be made by its manufacturer, is not guaranteed or endorsed by the publisher.

## Supplementary Material

Supplementary Figure 1 Spleen and liver masses in infected mice. Mice were infected with stationary-phase *L. donovani* in PBS and after 7d, were euthanized and the body weights determined. **(A)** Spleens and **(B)** livers were collected and individually weighted and used to calculate the organ mass by gram of total body weight. Graphs show individual mice (dots) and mean ± SEM of the combined datapoints from 3 independent experiments.

Supplementary Figure 2 Detection of LRV transcripts in *Leishmania*. Total RNA of stationary phase promastigotes from *L. donovani* MW897, *L. infantum* PP75 and *L. guyanensis* cultures LRV^high^ and LRV^low^ was extracted, the first strand cDNA synthesized and semi-quantitative PCR performed using LRV-specific primers.
