# Supplementary figures and images for: Toll-Like Receptor- and Protein Kinase R-Induced Type I Interferon Sustains Infection of Leishmania donovani in Macrophages
Source: Front Immunol. 2022 Jan 28;13:801182. doi: 10.3389/fimmu.2022.801182 (PMC8831251; doi:10.3389/fimmu.2022.801182)

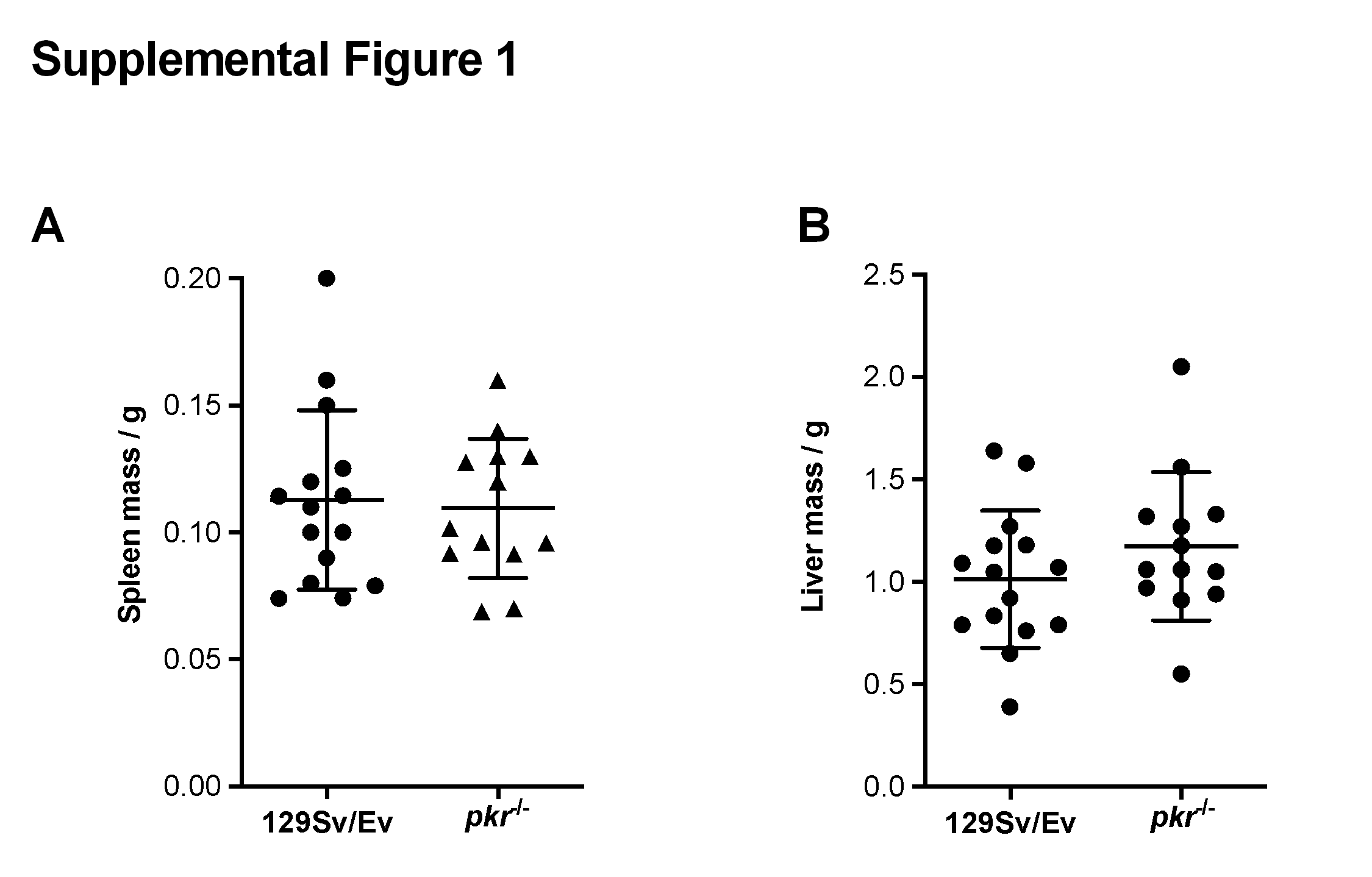

Supplement: Supplementary Figure 1 — Spleen and liver masses in infected mice. Mice were infected with stationary-phase L. donovani in PBS and after 7d, were euthanized and the body weights determined. (A) Spleens and (B) livers were collected and individually weighted and used to calculate the organ mass by gram of total body weight. Graphs show individual mice (dots) and mean ± SEM of the combined datapoints from 3 independent experiments. [file Image_1.tif]

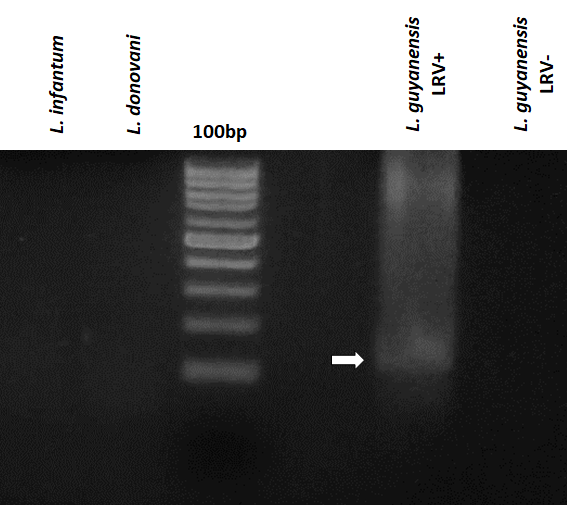

Supplement: Supplementary Figure 2 — Detection of LRV transcripts in Leishmania. Total RNA of stationary phase promastigotes from L. donovani MW897, L. infantum PP75 and L. guyanensis cultures LRVhigh and LRVlow was extracted, the first strand cDNA synthesized and semi-quantitative PCR performed using LRV-specific primers. [file Image_2.tiff]
