# Supplementary material for: Toll-Like Receptor- and Protein Kinase R-Induced Type I Interferon Sustains Infection of Leishmania donovani in Macrophages
Source: Front Immunol. 2022 Jan 28;13:801182. doi: 10.3389/fimmu.2022.801182 (PMC8831251; doi:10.3389/fimmu.2022.801182)
Supplement: Supplementary file 3 [file Table_1.docx]

**Supplementary Table 1**

**Sequence**

**Targets**

**IFN-β F**

**IFN-β R**

**IFN-α F**

**IFN-α R**

**IL-10 F**

**IL-10 R**

**SOD1-F**

**SOD1-R**

**OASL2 F**

**OASL2 R**

**PKR F**

**PKR R**

**GAPDH F**

**GAPDH R**

**5’-TCCAAGAAAGGACGAACATTCG-3’**

**5’-TGAGGACATCTCCCACGTCAA-3’**

**5’-CTGGCTGTGARRAMATACTTCC-3’**

**5’-TTCTGCTCTGACMACCTCC-3’**

**5’-CCCAGAAATCAAGGAGCATT-3’**

**5’-TCACTCTTCACCTGCTCCAC-3’**

**5’-GGCAAGCGGTGAACCAGTTGTGTT-3’**

**5’-TGAGGTCCTGCACTGGTACAGCC-3’**

**5’-CAAGAAGTCAGGGTGATTAAGG-3’**

**5’-GAACAGAATCATGTCTTGGTCA-3’**

**5’-GATGGAAAATCCCGAACAAGGAG-3’**

**5’-AGGCCCAAAGCAAAGATGTCCAC-3’**

**5’-TGCACCACCAACTGCTTAGC-3’**

**5’-GGCATGGACTGTGGTCATGAG-3’**

Table 1: Nucleotide sequence of primers used in qPCR assays. F, forward sense primer; R, reverse anti-sense primer.
